# Gender and Cultural Differences in the Relationships between Self-Esteem Contingency, Body Talk, and Body Esteem

**DOI:** 10.3390/children8111009

**Published:** 2021-11-04

**Authors:** Jounghwa Choi, Yoojin Chung, Hye Eun Lee, Michael Prieler

**Affiliations:** 1 Department of Advertising & Public Relations, Hallym University, Chuncheon 24252, Korea; 2 Department of Communication & Media, Ewha Womans University, Seoul 04315, Korea; chung.yoojin@ewhain.net (Y.C.); hyeeunlee77@ewha.ac.kr (H.E.L.); 3 Media School, Hallym University, Chuncheon 24252, Korea; prieler@hallym.ac.kr

**Keywords:** contingency of self-esteem, body talk, body esteem, gender, culture

## Abstract

This study analyzed the positive and negative body talk of male and female adolescents cross-culturally with an emphasis on the role of appearance-contingent and others’ approval-contingent self-worth. A cross-national survey in Austria, Belgium, Spain, and South Korea among 12- to 16-year-olds (982 female and 993 male) found that (1) positive body talk was positively related and negative body talk was negatively related to body esteem; (2) appearance contingency was positively related to negative body talk; (3) appearance contingency increased positive body talk among girls (except Korean girls); and (4) contingency on other’s approval increased positive body talk among boys in all four countries. Overall, gender differences were more prominent than cultural differences and positive body talk was instrumental in promoting adolescents’ body esteem.

## 1. Introduction

As body esteem is an important source of self-esteem among adolescents [1], they frequently engage in appearance or body-related talk. Nichter and Vuckovic conceptualized negative appearance-focused dialogue as “fat talk”, in which people share self-disparaging remarks about their bodies to each other [2]. In fact, studies have shown that most young females engage in fat talk [3], and this phenomenon is also common in other age groups [4]. Boys and young men have also been found to engage in such body-related conversations [5,6].

Previous literature has suggested that fat talk may negatively affect individuals’ well-being. Studies have documented that fat talk positively correlates with negative psychological outcomes such as perceived stress, drive for thinness, body dissatisfaction, and eating disorders [5,7,8,9,10,11]. Despite the accumulating literature regarding fat talk (for a review, see Mills and Fuller-Tyszkiewicz; Shannon and Mills [12,13]), the limitations in existing studies call for further exploration.

First, previous studies have primarily focused on fat talk (i.e., negative body talk), and relatively few studies [8,14] have explored the role of self-accepting (i.e., positive) body talk. Although self-degrading body talk tends to be perceived as more normative than self-accepting body talk [15], self-accepting body talk does exist [16]. Several studies have reported correlations of positive body talk with well-being outcomes [8,14]. Despite the suggested positive influences of self-accepting body talk, only a few studies have explored the roles of both negative and positive body talk with equal emphasis.

Second, while a number of studies have been conducted to explore the consequences of body talk, a relatively small number of studies have explored individual factors that affect it. In this regard, contingencies on self-worth (CSW) [17] might be a promising individual difference variable that affects body talk. While certain domains of CSW, i.e., appearance CSW and others’ approval CSW, have often been explored in relation to body image and body disturbances, no study has been found in relation to body talk. Exploring such relationships can be insightful for developing body esteem intervention programs that target a specific audience with customized contents based on a particular domain of self-worth contingency.

Finally, cross-gender and cross-cultural studies are scarce in the existing body talk research. The majority of body talk studies have been conducted among Western populations, with a few exceptions [18,19,20]. Scholars have also primarily focused on females, and studies that have explored body talk among males are still scarce (for exceptions, see Ahlich et al., Fiery et al. and Velkoff et al.’s studies [5,21,22]). Cultural contexts surrounding body talk can differ by gender and country, and such differences may also result in differences in the relationship among body talk, its predictors, and its consequences.

Shannon and Mills called for studies on fat talk with more diverse participants and investigated individual difference variables and cross-cultural variables [13]. Responding to this call, the present study aims to explore the relationships between contingent self-worth (i.e., self-worth contingent on appearance and others’ approval), body talk (i.e., negative and positive body talk), and body esteem among European and Korean adolescent females and males.

### 1.1. Body Talk and Body Esteem

Fat talk (or negative body talk) is the phenomenon in which women (or men) speak negatively about their weight, size, or shape of their bodies [2]. Negative body talk is common among all age groups and has become normative in society [4]. For example, research has shown that participants perceived it as more typical and less surprising if people engaged in negative rather than positive body talk [23]. Other research has also found that negative body talk is perceived as more believable than positive body talk, especially for overweight women [24]. Considering the prevalence of negative body talk in society and in social media, researchers have become concerned about its potential impact.

There have been numerous studies on body talk, and particularly negative body talk (i.e., fat talk) and its potential effects in recent years. Most studies have reported a relationship between negative body talk and body dissatisfaction [7,8,11,25,26,27]. Compeau and Ambwani, for example, found that negative body talk increased body dissatisfaction [26]. A meta-analysis of 24 studies by Sharpe et al. confirmed the positive relationship between negative body talk and body dissatisfaction, although this was true only for long-term and not for short-term studies [9]. Studies have also shown relationships between negative body talk and other body-related variables: negative body talk predicted higher depression levels and a perceived sociocultural pressure to be thin [25]; fat talk was positively correlated with body surveillance [28] and a drive for thinness [11]; frequency of negative body talk was positively related to eating disorders [5,7]; and a negative relationship was found between body talk and self-esteem [8]. Such previous findings suggest that negative body talk will negatively affect one’s body esteem.

In contrast to the increasing literature on negative body talk over the past years, there are comparatively few studies that have focused on positive body talk. For example, Rudiger and Winstead found that positive body talk is related to body satisfaction and self-esteem [8]. Similarly, Cruwys, Leverington, and Sheldon have shown that, for positive body talk, none of the negative effects of fat talk (i.e., negative body talk) applied, including disordered eating [14]. Although the empirical evidence is not ample enough, such findings suggest a positive relationship between positive body talk and body esteem. Therefore, we formulated the following hypotheses.

**Hypothesis** **1** **(H1).** *Positive body talk (H1-1) is positively related to body esteem, and negative body talk (H1-2) is negatively related to body esteem.*

### 1.2. Self-Worth Contingencies and Body Talk

The concept of contingencies of self-worth (CSW) states that individuals differ in the domains on which they base their self-worth [29]. For some people, self-esteem may depend on being loved, and for others, it may depend on looking attractive. Therefore, CSW researchers argue that understanding self-esteem as a whole could be too simplistic, and the suggested social or psychological issues due to low self-esteem in the previous literature have been overemphasized [30]. They have further argued that individuals’ behavior is better and more reliably understood by taking into account domain-based self-esteem rather than global self-esteem. Because people have a strong need to protect and enhance their self-esteem, contingencies of self-esteem are expected to serve as an important driver for an activity or behavior, which are relevant to the domains where people stake their self-worth [29]. Crocker and colleagues actually found that CSW domains were differentially related to types of behavior, i.e., time spent participating in different activities [31]. This result suggests that considering the relevant domain of CSW is important in predicting certain behavior and its consequences.

Scholars have identified seven domains of CSW: (1) approval from generalized others; (2) appearance; (3) competition; (4) academic competence; (5) family support; (6) virtue; and (7) God’s love [17]. Out of seven domains of self-worth contingency, the dimensions of appearance and others’ approval are relevant to the context of body esteem and body talks.

Appearance CSW concerns self-evaluations of one’s physical appearance. People, in particular females, are often evaluated on the basis of physical appearance [32] and thus learn to evaluate themselves in terms of physical appearance. It has been shown that people’s global self-esteem is significantly related to the evaluation of their own physical appearance, particularly among adolescents [33]. Because appearance CSW directly deals with the domain of body image, it is related to various body-related psychological and behavioral outcomes. Studies have shown that when people’s self-esteem is more contingent on appearance, they are more likely to exhibit body surveillance, body shame, and lower body esteem [34,35,36].

CSW based on approval from generalized others (others’ approval CSW) is when one’s self-esteem is based on receiving approval and acceptance from others. The views of others are deemed critical for overall CSW [37] and global self-esteem [38]. Compared to appearance CSW, others’ approval CSW has received less scholarly attention in relation to body image. However, several studies have reported that it is positively related to body dissatisfaction, body appreciation, and eating disorders [36,39]. For example, investing one’s self-esteem in others’ approval significantly reduces appearance satisfaction [36] and increases body appreciation and eating disorders [39].

Given the evidence from previous studies that suggest appearance CSW and others’ approval CSW are related to various body-related behavioral and psychological outcomes, this study expects that they will also influence adolescents’ body talks. To date, no known study has explored the role of CSWs in fat talk or body talk. However, given that CSWs have important implications for self-regulation thus influencing choice of course of action (for a review, see Crocker et al. [40]), CSWs, in particular appearance CSW and others’ approval CSW, may influence body talk.

It appears natural to expect appearance CSW to be related to body talk. Those who invest their self-esteem in appearance are likely to be concerned about their appearance and engage in body surveillance [34,36]. They are also more likely to experience greater body dissatisfaction and body shame [4,35,36]. Previous studies have shown that such body disturbance constructs are associated with negative body talk. For example, Aubrey and colleagues’ study reported that as women self-objectify themselves, they were more likely to negatively describe their body [41]. Arroyo and Harwood also found that low body satisfaction predicted more fat talk [25]. In summary, those who are high in appearance CSW are more likely to be concerned about their body and subsequently engage in body talk, as body talk can often offer reassurance [3].

Others’ approval CSW is also expected to be related to body talk. Although it has rarely been discussed in relation to body image, ample evidence that conformity influences body talk suggests such a relationship. Previous research suggests that body talk, in particular fat talk, is deemed a normative behavior [4,42] and often serves as a social control tool [43]. Women engage in fat talk because they see it as socially expected behavior or feel pressure to do it from the group [2,43,44]. Such findings suggest that females utilize fat talk to gain social acceptance by a valued group. Therefore, when body talk is normative, adolescents who are more conscious of others (i.e., high on others’ approval CSW) are likely to engage in body talk to a greater extent and feel afraid of social ostracism by failing to do it.

Although both appearance CSW and others’ approval CSW are expected to be related to body talk, most previous research concerns fat talk, i.e., negative body talk. Therefore, it is not clear how those CSW are related to positive body talk. Appearance CSW and others’ approval CSW might relate differentially to positive and negative body talk. For example, CSW might be more related to negative body talk than positive body talk because those whose self-worth depends on appearance may have more significant body concerns and may thus engage in more negative body talk, as suggested in previous fat talk research. On the other hand, others’ approval CSW might be more related to positive body talk because those who are highly concerned about others may have a stronger need for impression management.

Given the limited empirical evidence to support the aforementioned relationships, the following research questions (RQ) are put forth:

**RQ1.**  *How is appearance-contingent self-worth related to body talk [i.e., (a) negative and (b) positive body talk]?*

**RQ2.**  *How is others’ approval-contingent self-worth related to body talk [i.e., (a) negative and (b) positive body talk]?*

### 1.3. The Role of Gender and Culture

While there is some evidence about the possible impacts of body talk on self-esteem, comparatively little is known about the role gender might play in this context. The main reason for that is that the majority of studies have focused on women considering the pervasiveness of body talk among females. Indeed, a couple of studies that examined gender differences in body talk have reported that women are more likely to hear and participate in fat talk [16,45]. However, a few studies also reported that body talk can play a role in men’s well-being. A study reported that for men, negative body talk was related to a drive for muscularity, eating disorders, and appearance investment but also to decreased appearance self-esteem and body dissatisfaction [6]. Another study on gay and heterosexual men found that gay men more frequently engage in body talk and have greater body dissatisfaction than heterosexual men [46]. Such findings suggest that body talk is also a relevant issue among men and that there is still much research that needs to be completed on gender differences and body talk, since only a few studies considered gender as a variable and some have found no gender differences [28].

Across all countries, women are evaluated in terms of their physical appearance and attractiveness [47]. In such a social context, it might be natural that women engage in body talk to a greater extent, and the consequences of body talk might be more pronounced among women. Additionally, the relationships of appearance CSW and others’ approval CSW with body talk might differ by gender. For example, previous studies have suggested that women tend to think of conformity as positive and self-defining, while men define themselves when they are not conforming [48]. Therefore, women whose self-esteem is based on others’ approval may engage in body talk to a greater extent, as they can better sense social norms about the body and appearance through such conversation. Similarly, as women tend to be high in appearance CSW [31], appearance CSW may play a more active role in body talk initiation among women than men.

Similar to the case of gender differences, there is very little known about cultural differences in body talk, since the majority of studies have been conducted in Western countries (for exceptions, see Chen et al.’s and Wang, Yang et al.’ studies [10,49]), and these studies have been mostly single-country studies. Among the few cross-cultural studies, two cross-cultural studies included non-Western countries: Taniguchi and Lee compared exposure to fat talk in Facebook in Japan and the US [20], and Lee et al. compared exposure to fat talk in Facebook in South Korea and the United States [18]. Both studies manipulated Facebook posts (body size of profile owner, underweight vs. overweight, message from peers, weight loss encouragement vs. discouragement). Lee et al.’s study found that in South Korea, peers had a larger influence on the outcomes of body talk than in the United States [18]. For example, in South Korea, it mattered whether peers on Facebook were underweight or overweight, and it also mattered whether peers posted messages discouraging weight loss, while this was not the case in the US. Similarly, Taniguchi and Lee found that thin-promoting messages led to lower body satisfaction only in Japan, but thin discouraging messages led to better well-being among both Americans and Japanese females [20]. Both studies indicate that peers might play a more important role in Japan and Korea than in the United States.

These findings might be in accordance with cultural differences between these cultures. Hofstede’s individualism and collectivism seem particularly related to this phenomenon [50]. According to Hofstede and associates, in individualistic societies, the ties between individuals are loose, while in collectivistic society, people are integrated into strong, cohesive in-groups [51]. The self is construed as unique and independent in individualistic cultures, while people tend to construe themselves in connection with the social context in collectivistic cultures [52]. Research has shown that people with higher collectivistic scores (such as South Koreans) are more likely to perform social comparison [53,54] than people with lower collectivistic scores. This previous research suggests that Asians and Europeans may differ in the extent to which they base their self-esteem in others’ approval and that the influence of others’ approval might be more pronounced among Asians.

As discussed above, previous research implies gender and cross-cultural differences in the aforementioned relationships. Given the limited previous studies, we formulated the following research questions:

**RQ3.**  *Do the aforementioned relationships differ by gender (RQ3-1) or country (RQ3-2)?*

## 2. Method

### 2.1. Participants

The study utilized data from a large-scale cross-national survey conducted in Austria, Belgium, Spain, and South Korea in 2017 to investigate media use among adolescents (12 to 16 years old) and its influence on well-being. Table 1 shows the sample size and age description of each country. The selection of countries was based on their differences on the individualism–collectivism dimension of Hofstede and associates [51]. Belgium scored 75, Austria 55, and Spain 51; South Korea was the least individualistic (and most collectivistic) culture with a score of 18. Due to the difficulty in obtaining permission from the schools, a convenient sampling was employed. The host investigators of each country contacted schools in each country by writing letters or making phone-calls, and sometimes through their own personal acquaintances. Seven schools in Austria, eleven schools in Belgium, four schools in South Korea, and five schools in Spain agreed to participate. Recruitment notices along with the informed consent forms were distributed to the students before the survey was conducted (in some schools, a limited number of students were given access, such as specific classes in each grade). Students who brought their own and their parents’ consent to participate were allowed to take the survey during or after school hours under the researchers’ supervision. A total of 982 female and 993 male adolescents completed the questionnaire. This study was approved by the ethics committees of each host university.

### 2.2. Instrument and Measures

The questionnaire was initially constructed in English and then translated into the researchers’ native languages (German, Dutch, Spanish, and Korean) under the same guidelines (translation, back-translation, and review by bilingual researchers).

The dimensions of others’ approval and appearance contingency of self-worth were measured by utilizing Crocker et al.’s self-worth scale [31]. Three items were used for each measure after the confirmatory factor analysis (for others’ approval contingency: “I don’t care what other people think of me,” “What others think of me has no effect on what I think about myself,” and “I don’t care if other people have a negative opinion about me”; for appearance self-worth: “My self-esteem is influenced by how attractive I think my face or facial features are,” “My sense of self-worth suffers whenever I think I don’t look good,” and “When I think I look attractive, I feel good about myself”). These variables were measured with 7-point Likert scales (1 = “strongly disagree” to 7 = “strongly agree”).

The measure of body talk was adapted from previous [8] and was split into body talk positive and body talk negative in everyday life. Body talk positive included the following four items: “Imagine you and your friends saying positive things about your/their own bodies or appearances (for example, ‘I look good today’ or ‘I really like my hair’). How often does this occur?”; “How often do you say positive things about your own body or appearance in front of your friends?”; “How often do you receive/overhear positive comments about your own body or appearance?”; and “How often do your friends say positive things about their own bodies or appearance in front of you”? For body talk negative, the same items were used but were changed to negative statements, for example, “How often do you say negative things about your own body or appearance in front of your friends”? This variable was measured with a 5-point Likert scale asking how often participants engaged in such conversation (1 = “never” to 5 = “very frequently”).

The variable body esteem was measured with a shortened version of the Body Esteem Scale for Adolescents and Adults [55]. For acceptable unidimensionality and reliability across cultures, seven items were used in the survey, including “Other people consider me good looking”, “I like what I see when I look in the mirror”, “I am satisfied with my weight”, “I really like what I weigh”, “I’m pretty happy about the way I look”, “I think I have a good body”, and “I’m looking as nice as I’d like to”. This variable was measured with a 5-point Likert scale asking the extent to which participants agreed with the statements (1 = “never” to 5 = “always”).

Along with these key variables, media use (i.e., time spent watching TV and reading magazines) was measured and was included as a control variable in the analysis. Participants were asked to indicate how many minutes per day they spent watching TV and reading magazines in general (1= “never use it less than 10 min” to 7 = “>6 h”). These two items were averaged. Additionally, body mass index was considered in the analysis. The descriptive statistics of the key variables and their correlations are presented in Table 2.

Multigroup structural equation modeling was conducted to test the proposed model using Mplus 8.0. Groups were divided by gender and country, so eight groups were compared. To evaluate the model fit, the following criteria were used: CFI (confirmatory fit index), TLI (Tucker–Lewis index), and RMSEA (root mean square error of approximation).

## 3. Results

**Figure 1:**
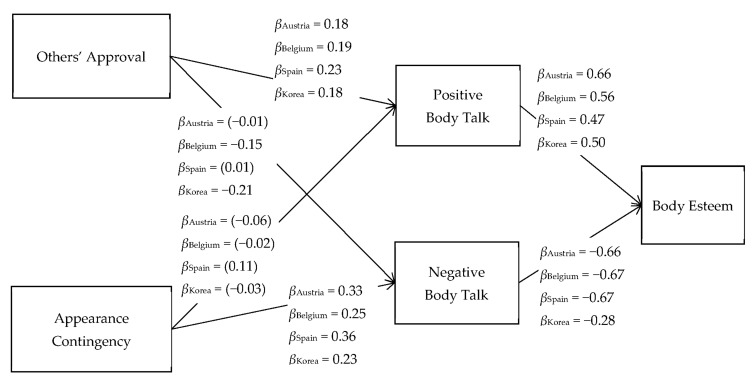
The final model of the relationships between others’ approval, appearance contingency, positive and negative body talk, and body esteem among boys. Note that the values are the observed standardized path coefficients. The path coefficients without parentheses are significant at *p* < 0.05.

**Figure 2:**
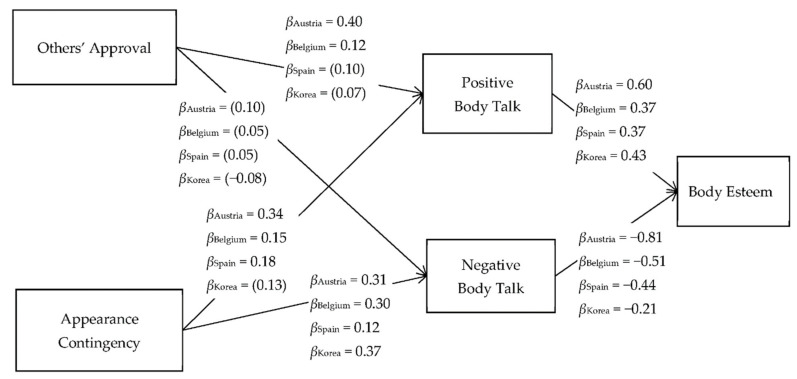
The final model of the relationships between others’ approval, appearance contingency, positive and negative body talk, and body esteem among girls. Note that the values are the observed standardized path coefficients. The path coefficients without parentheses are significant at *p* < 0.05.

Acceptable goodness-of-fit indices were obtained for the overall model (χ2(df) = 5461.0(1680), *p* < 0.01, CFI = 0.97, TLI = 0.94, RMSEA = 0.05) and each group (CFI = 0.95~0.98, TLI = 0.94~0.98, RMSEA = 0.03~0.05) [56]. The estimated coefficients are presented in Figure 1 and Figure 2.

### 3.1. The Relationship between Body Talk and Body Esteem

Results consistent with the hypotheses (H1-1 and H1-2) were found for every group regardless of gender and nationality. All boys and girls showed significant positive effects of positive body talk (β = 0.37 to 0.66) and negative effects of negative body talk (β = −0.21 to −0.81) on body esteem.

### 3.2. Cross-National Comparisons of Relationships of Others’ Approval and Appearance Contingency of Self-Worth with Positive and Negative Body Talk (RQ1, RQ2, and RQ3-2)

Among boys, regardless of country, others’ approval contingency significantly and positively affected positive body talk (β = 0.18 to 23). In contrast, boys’ appearance contingency did not influence positive body talk. Others’ approval contingency did not affect negative body talk among Austrian and Spanish boys but did have such an effect on Belgian (β = −0.15) and Korean (β = −0.21) boys. On the other hand, appearance contingency positively affected negative body talk for boys regardless of nationality (β = 0.23 to 0.36).

Among Spanish and Korean girls, others’ approval contingency did not affect positive body talk, whereas it positively influenced positive body talk for Austrian (β = 0.40) and Belgian (β = 0.12) girls. Additionally, this approval contingency did not affect negative body talk among girls across countries. In contrast, appearance contingency significantly and positively influenced positive body talk among girls (β = 0.15 to 0.34), except in Korea, where there was no relationship between appearance contingency and positive body talk. Finally, across the countries, girls’ appearance contingency significantly and positively affected negative body talk (β = 0.12 to 0.37).

### 3.3. Gender Differences among the Relationships (RQ3-1)

Gender differences were found in the relationships between appearance contingency and positive body talk in European countries (Austrian boys: β = −0.06 vs. girls: β = 0.34; Belgium boys: β = −0.02 vs. girls: β = 0.15; Spanish boys: β = 0.11 vs. girls: β = 0.17), in the relationship between others’ approval and positive body talk in Spain (boys: β = 0.23 vs. girls: β = 0.10) and Korea (boys: β = 0.18 vs. girls: β = 0.07), and in the relationship between others’ approval and negative body talk in Korea (boys: β = −0.21 vs. girls: β = −0.08).

## 4. Conclusions

This study examined the relationships between two domains of CSW (i.e., appearance and others’ approval), two types of body talk (i.e., positive and negative), and body esteem among young adolescents with cultural comparisons and gender comparisons.

Summarizing the key findings of this study, as proposed in Hypothesis 1, positive body talk was positively related to body esteem, whereas negative body talk was negatively related to body esteem across countries and genders. This finding is consistent with Cruwys et al. and Rudiger and Winstead, who found that positive/self-accepting body talk led to positive psychological outcomes, whereas negative body talk led to body disturbances [8,14]. This result shows that sharing positive body talk makes adolescents more confident about their bodies and allows them to receive peer support, whereas negative body talk plays the opposite role.

In exploring the relationship between appearance CSW and body talk (RQ1), this study found a positive relationship between appearance CSW and negative body talk across countries and genders. This result might be because adolescents whose self-worth relies on appearance have greater body image concerns [34], and thus engage in negative body talk. Unlike for boys, the appearance CSW for girls, except Korean girls, increased not only negative body talk but also positive body talk. Therefore, it appears that appearance CSW plays a more active role among European girls than among European boys. This finding is not surprising given that women are pervasively evaluated by others in terms of physical appearance and attractiveness [47]; thus, women scored higher on appearance CSW [31], and engaging in self-degradation is a norm among women [15].

On the other hand, exploring the relationship between others’ approval CSW and body talk (RQ2), this study found that others’ approval CSW increased positive body talk regardless of their country among boys. It appears that boys who were more concerned about others engaged in positive body talk more, possibly because of impression management motives. This finding is in line with previous studies that have reported that men are more likely to engage in positive body talk [6,16] and tend to report more pressure to engage in positive body talk [45].

Finally, in exploring the difference by gender (RQ3-1) or country (RQ3-2), this study found that the relationship between body talk and body esteem was consistent across countries and genders. Such a pattern also emerged in the relationship between appearance CSW and negative body talk. On the other hand, gender difference was found in the relationship between the appearance CSW and positive body talk as mentioned in discussing the results for RQ1. In the relationship between others’ approval CSW and positive/negative body talk, gender difference rather than cultural difference emerged: others’ approval CSW was positively related to positive body talk regardless of countries among boys whereas such a relationship was found in only two countries among girls; others’ approval CSW was not related to negative body talk across countries among girls whereas it was related in two countries among boys. 

To conclude, even though some differences by country were found in the explored relationships, there was no clear pattern of differences between European countries and Korea; thus, the difference between boys and girls appears to be more prominent than a cultural difference.

This study has theoretical implications in that it shows that both types of body talk (i.e., negative and positive) contribute to body esteem. While the majority of previous studies have focused on the negative consequences of negative body talk, the observed positive influence of positive body talk suggests that such talks can be instrumental in promoting adolescents’ body esteem. This study also showed that not only appearance CSW but also others’ approval CSW is an important driver of body talk. Although their relationships with body talk somewhat varied by country and gender, appearance CSW tended to be more related to body talk among girls than among boys, and others’ approval CSW tended to be more related to body talk among boys than among girls. As such gender differences in the role of CSW domains were found, future studies that explore body talk and body images should consider these different domains of CSW considering their target population of research.

This study also offers practical implications. Given the positive influence of positive body talk on body esteem, it might be beneficial for a body esteem educational program to teach adolescents how to share appropriate and positive comments about their bodies with friends. In addition, as appearance CSW and others’ approval CSW were differentially related to body talk depending on gender, a body esteem educational program may need to approach girls and boys with different messages that focus on different domains of self-esteem contingency (i.e., appearance contingency for girls and others’ approval contingency for boys). For example, messages targeting girls may need to address the adverse effects of investing their own self-esteem in appearance; messages targeting boys may need to address the value of listening to their own voice rather than others. Such an approach with customized contents addressing a particular domain of self-worth contingency will allow for more effective intervention programs. Research has shown that intervention programs can in fact reduce fat talk frequency and weight concerns [57].

This study has several limitations that can be examined in future research. First, the study participants were not well balanced in terms of culture: they came from three European countries and one Asian country. Therefore, while this study examined cross-national differences, it is unclear what cultural factors were operating and driving the differences by country. As a result, future studies might include a more balanced sample in terms of geographical distribution, potentially also understudied regions such as Africa. Second, because the data used in this study were cross-sectional in nature, causal relationships cannot be claimed. It is possible that those who have low body esteem are more engaged in negative body talk and less engaged in positive body talk. A longitudinal or experimental research design will be beneficial to draw definitive conclusions for the causality of the relationships examined in this study. In particular, an experimental study that tests the effect of an intervention program designed to influence appearance CSW and others’ approval CSW would be beneficial.

In conclusion, while the negative consequences of negative body talk have been emphasized in previous studies, the role of positive body talk has received little academic attention. In addition, the exploration of individual factors that influence body talk has been limited, as have gender differences or cultural differences in relation to body talk. By addressing these limitations in the existing literature, the present study extends the literature on body talk and provides practical implications for body image intervention.

## Figures and Tables

**Table 1:** Description of the participants.

	Number of Participants	M of Age	SD of Age
Austria			
Boy	198	16.07	1.21
Girl	170	16.11	1.25
All	368	16.09	1.23
Belgium			
Boy	291	14.70	0.67
Girl	380	14.74	0.71
All	671	14.72	0.69
Spain			
Boy	306	16.06	0.84
Girl	252	16.03	0.87
All	559	16.04	0.86
Korea			
Boy	187	14.96	0.84
Girl	191	15.03	0.81
All	378	15.00	0.82
Total			
Boy	982	15.46	1.10
Girl	993	15.34	1.06
All	1976	15.41	1.08

M: Mean, SD: Standed Deviation.

**Table 2:** Reliabilities, Correlations, Means, and Standard Deviations.

**Boys**	**OA**	**AC**	**PBT**	**NBT**	**BE**	**M**	**SD**
Austria							
Others’ Approval Contingency	(0.84)					4.80	1.48
Appearance Contingency	−0.31 **	(0.68)				4.03	1.26
Positive Body Talk	0.27 **	0.06	(0.79)			1.90	0.67
Negative Body Talk	−0.09	0.29 **	0.16	(0.56)		2.42	0.84
Body Esteem	0.31 **	−0.26 **	0.33 **	−0.34 **	(0.90)	3.24	0.95
BMI	−0.01	0.05	−0.13 **	−0.01	−0.37 **	19.77	3.53
Belgium							
Others’ Approval Contingency	(0.84)					4.49	1.32
Appearance Contingency	−0.24 **	(0.65)				3.81	1.13
Positive Body Talk	0.13 **	0.07	(0.74)			2.33	0.80
Negative Body Talk	−0.15	0.26 **	0.18 **	(0.76)		1.99	0.77
Body Esteem	0.40 **	−0.18 **	0.18 **	−0.37 **	(0.90)	3.50	0.89
BMI	0.05	0.13 **	0.22 **	0.23 **	−0.21 **	19.78	3.53
Spain							
Others’ Approval Contingency	(0.83)					4.60	1.40
Appearance Contingency	−0.37 **	(0.66)				3.74	1.21
Positive Body Talk	0.10 **	0.15	(0.81)			2.41	0.82
Negative Body Talk	−0.18 **	0.37 **	0.23 **	(0.69)		1.97	0.69
Body Esteem	0.26 **	−0.19 **	0.22 **	−0.35 **	(0.89)	3.43	0.90
BMI	0.03	−0.03	−0.01	0.02	−0.12 **	20.01	3.25
Korea							
Others’ Approval Contingency	(0.91)					3.97	1.38
Appearance Contingency	−0.13 **	(0.65)				4.04	1.08
Positive Body Talk	0.12 **	0.11	(0.87)			2.71	0.96
Negative Body Talk	−0.24 **	0.30 **	0.17 **	(0.89)		2.05	0.94
Body Esteem	0.32 **	0.03	0.33 **	−0.23 **	(0.87)	2.79	0.85
BMI	0.08	0.01	−0.04	−0.01	−0.24 **	20.28	3.25
**Girls**	**OA**	**AC**	**PBT**	**NBT**	**BE**	**M**	**SD**
Austria							
Others’ Approval Contingency	(0.84)					4.80	1.48
Appearance Contingency	−0.31 **	(0.68)				4.03	1.26
Positive Body Talk	0.27 **	0.06	(0.79)			1.90	0.67
Negative Body Talk	−0.09	0.29 **	0.16	(0.56)		2.42	0.84
Body Esteem	0.31 **	−0.26 **	0.33 **	−0.34 **	(0.90)	3.24	0.95
BMI	−0.02	0.05	−0.13**	−0.13	−0.36**	20.76	3.62
Belgium							
Others’ Approval Contingency	(0.84)					4.49	1.32
Appearance Contingency	−0.24 **	(0.65)				3.81	1.13
Positive Body Talk	0.13 **	0.07	(0.74)			2.33	0.80
Negative Body Talk	−0.15	0.26 **	0.18 **	(0.76)		1.99	0.77
Body Esteem	0.40 **	−0.18 **	0.18 **	−0.37 **	(0.90)	3.50	0.89
BMI	0.08	0.10	0.27**	0.24 **	−0.18 **	18.69	4.38
Spain							
Others’ Approval Contingency	(0.83)					4.60	1.40
Appearance Contingency	−0.37 **	(0.66)				3.74	1.21
Positive Body Talk	0.10 **	0.15	(0.81)			2.41	0.82
Negative Body Talk	−0.18 **	0.37 **	0.23 **	(0.69)		1.97	0.69
Body Esteem	0.26 **	−0.19 **	0.22 **	−0.35 **	(0.89)	3.43	0.90
BMI	−0.01	−0.07	−0.04	0.01	−0.08	20.11	3.20
Korea							
Others’ Approval Contingency	(.91)					3.97	1.38
Appearance Contingency	−0.13 **	(0.65)				4.04	1.08
Positive Body Talk	0.12 **	0.11	(0.87)			2.71	0.96
Negative Body Talk	−0.24 **	0.30 **	0.17 **	(0.89)		2.05	0.94
Body Esteem	0.32 **	0.03	0.33 **	−0.23 **	(0.87)	2.79	0.85
BMI	−0.01	0.10	−0.03	0.08	0.36 **	20.65	3.71

** *p* < 0.01. Reliabilities are reported in parentheses on the diagonal. Others’ approval (OA); appearance contingency (AC); positive body talk (PBT); negative body talk (NBT); body esteem (BE), and body mass index (BMI). The items for others’ approval contingency and appearance contingency were measured a 7-point Likert scale, and the other items were measured on a 5-point Likert scale.

## Data Availability

Qualified researchers can obtain the data from the corresponding author (jhchoi@hallym.ac.kr). The data are not publicly available due to privacy concerns imposed by the IRB.
